# Integrated Care Regulation, Assessment, and Inspection – A Collaborative Learning Journey

**DOI:** 10.5334/ijic.4677

**Published:** 2019-03-25

**Authors:** Patricia Sullivan-Taylor

**Affiliations:** 1 Policy and Partner Engagement, Health Standards Organization, Ottawa, CA

**Keywords:** integrated care systems, regulation, assessment and inspection, standards, person centred, population health, quality improvement

Over the past ten years, the design and implementation of integrated health and social care organisations and systems has occurred across several continents. These models of care have implied the adoption of new approaches to their organization and governance. As a result, many countries have begun to focus specifically on the regulation, assessment and inspection of integrated care. This editorial argues that there is both an opportunity and a need to share the successes and challenges from these experiences and to promote methods for collaborative learning.

Three themes appear to be driving and influencing the focus of regulation, assessment and inspection when it comes to integrated health and social care:

1. First, advancing integrated care within health and social services is an international priority. People are living longer and have increased rates of chronic, long-term conditions and comorbidities. Policy makers must balance sustainable economic growth with population changes. These changes increase demand and reinforce the need for integrated services that are based on strong primary care and public health function [1]. For example, the English NHS has outlined in its ten year plan that it expects local areas to move towards integrated care systems by 2019. This commitment includes more coordinated care aimed at health promotion and prevention, as well as service differentiation based on individual needs [2].
  Concurrently, governments must improve health and equity while efficiently managing costs and accountability. A 2018 external review of the Pan-Canadian Health Organizations reinforced the need for strategic partnership between federal governments, provinces and territories “to accelerate comprehensive, integrated publicly funded health systems centred in primary care.” It further promoted the need for “spread and scale of system innovations, using all levers, including policy and regulation” [3].
  Regulation is designed to protect and serve the public interest by ensuring transparent and accountable systems, while establishing expected outcomes. Assessments and inspections are commonly used to monitor the degree to which regulations or standards are being met across health and social systems [4]. Assessment and inspection of integrated care is meant to improve patient safety, foster a quality culture and monitor the effectiveness of programs [5,6].
2. Second, while recognized as a global priority, integrated care development and implementation is in the early stages in most countries; some struggle to move beyond the pilot phase [7]. Integrated care, like any major system transformation, requires levers at the system (macro), organizational (meso) and care (micro) level. Assessment and inspection within and across these inter-dependent levels help governments, administrators and providers monitor what is working, as well as opportunities to continually improve care. As such, the role of regulation, assessment and inspection complement key elements of a learning health system [8,9]. Learnings from assessments and inspections inform refinement and support needed to scale broadly across system, organizational and care levels.
  In Scotland substantial progress has been made regarding the introduction of legal structures and governance arrangements that enable more integrated care. It still faces implementation challenges to scale at the care-level [10]. There is opportunity to learn from one another about the enablers and barriers to sustain and augment successful integrated care models. Historical system transformation efforts demonstrate the need for approaches that combine a clear vision, co-designed with key stakeholders, with bottom-up and top-down implementation enablers. Furthermore, advancing integrated people-centred systems requires long-term policy commitment and investment, leadership across the health and social care systems supported by integrated information systems [11,12].
3. Third, though most countries are in early stages of design, a single model toward regulating and inspecting integrated care is unlikely to work. Adaptation across context, settings and countries is needed [13]. Integrated care does not lend itself to a universal, one-size fits all approach. Lessons from Canada, Scotland and England have reinforced that standards and regulations are useful to set expectations on outcomes. However, local administrators and providers must be empowered to implement with flexibility based on local needs and context [14,15]. Consequently, various models, assessment frameworks, policy enablers and implementation resources continue to evolve.
  Regulation, assessment and inspection programs must be effective, efficient and responsive at fostering quality improvement and compliance while limiting burden for organizations and systems [16]. Identified barriers include misaligned regulation and/or legislation [17]. Even countries with enabling legal obligations and national initiatives “still struggle with establishing sustainable integrated care…without the multidisciplinary teamwork needed across organizational boundaries” [18].
  Furthermore, integrated care payment models need to incentivize cooperation rather than competition and ensure that “payment is contingent on quality and outcome targets…across a whole system” [19,20]. Governments and administrators will need to evolve regulation, assessment and inspection programs as the integrated systems mature and evidence on what works becomes more understood.

In Canada, Health Standards Organization (HSO) is responding to these needs through co-designing standards and implementation resources, in partnership with patients, providers, administrators and policy makers. In 2018, HSO drafted an Integrated People Centred Health and Social Services Standard designed for authorities and jurisdictions (province, state, region, sub-region or municipal-level) [21]. The standard includes evidence-based criteria and guidelines to assist decision-makers as they plan, design, implement and evaluate integrated health and social service systems. The aim is to support clients with quality improvement and assessment, rather than regulation or inspection [22].

HSO conducted a three-month public review of the draft standard, as well as targeted consultation with patients, providers, policy makers, existing Accreditation Canada health system clients and international experts. This input will inform the final standard and implementation resources. It will enable clients across 30 countries to deliver better population health outcomes, improved care experiences, client and provider engagement at manageable costs. The final HSO 90000 Integrated People Centred Services Standard will be published in 2019. Key themes that emerged from consultation will also be published in 2019.

These HSO research findings will also be shared within the newly launched Regulating and Inspecting Integrated Care Special Interest Group (RIIC-SIG) that is supported by IFIC. This global group will debate observations, solutions and challenges related to regulating, assessing and inspecting integrated care. The RIIC-SIG brings together professionals representing organizations that operate as regulators, inspectors, auditors or quality assessors in the health and social domain. The group initially represented more than 10 countries at the IFIC 2018 Spring Conference in Utrecht, where areas of interest, challenges and initiatives underway were shared. A workshop was convened in Edinburgh in October 2018 to finalize the Terms of Reference for the RIIC-SIG.

RIIC-SIG objectives for 2019–2021 include:

1. Establishing a platform to share relevant material such as frameworks, experiences, leading practices and approaches (2019);
2. Identifying and securing the necessary resources to ensure sustainability of the RIIC-SIG (2019);
3. Developing and publishing a paper on assessing, regulating and inspecting integrated care (2020);
4. Defining building blocks for regulating/inspecting integrated care and common research questions on regulating/inspecting integrated care (2020);
5. Establishing relationships with researchers to stimulate research (2020–2021); and
6. Evaluating the outcome and impact of the RIIC-SIG (2021).

The RIIC-SIG will meet at IFIC 2019 in San Sebastien. A workshop hosted by HSO is being planned for IFIC 2020 in Toronto. For more information on becoming a RIIC-SIG member, please contact Heleen Buijze ph.buijze@igj.nl, Kees Reedijk cj.reedijk@igj.nl or Janet Ortega janet.ortega@cqc.org.uk.

## Author Information

Executive Lead, Policy and Partner Engagement, Health Standards Organization, Ottawa, Canada.
